# Review of the Effects of Antiviral Therapy on Hepatitis B/C-Related Mortality and the Regression of Fibrosis

**DOI:** 10.3390/v16101531

**Published:** 2024-09-27

**Authors:** Stephen Sinclair, Sean Shearen, Youssef Ghobrial, George Trad, Syed Abdul Basit, David Shih, John K. Ryan

**Affiliations:** Comprehensive Digestive Institute of Nevada, Las Vegas, NV 89148, USAsab@nevadagastro.com (S.A.B.); dshih@nevadagastro.com (D.S.)

**Keywords:** Hepatitis B, Hepatitis C, HCV, HBV, cirrhosis, fibrosis, decompensated cirrhosis, viral Hepatitis, liver-related mortality, portal hypertension

## Abstract

Hepatitis B and Hepatitis C are viral causes of Hepatitis that lead to significant worldwide mortality and morbidity through the sequelae of fibrosis and hepatocellular carcinoma. In this review, we have summarized recent studies that have examined the effects of antiviral therapy on the regression of fibrosis and the reduction in mortalities associated with the viruses. Antiviral therapy significantly decreases mortality and induces the regression of fibrosis.

## 1. Introduction

### 1.1. General

Hepatitis C and Hepatitis B are important viruses to virologists, biomedical researchers, healthcare providers, and to the public. The viruses cause significant mortality and the fibrosis of the liver. This review summarizes the literature which specifically addresses the reduction in mortality and the regression of fibrosis induced by antiviral therapy. The remainder of the introduction will focus on the specific viruses’ history, epidemiology, virology, and clinical manifestations.

### 1.2. Hepatitis C

The Hepatitis C virus (HCV), identified in 1989 following nearly 20 years as an unidentified Hepatitis-inducing agent in post-blood transfusion patients, remains a target for elimination as a public health threat by the World Health Organization (WHO) by 2030 [1]. Estimates indicate a total global prevalence of Hepatitis C at 2.5%, and viremic or active infections range from 0.7%, or 56.8 million people, to as high as 80 million people [1,2,3,4]. Geographic locations such as Asia, Eurasia and western Europe, and parts of Africa tend to have the highest rates of Hepatitis C, with China, Pakistan, Nigeria, Egypt, India, and Russia alone accounting for over half of total global infections [4]. Furthermore, local areas with higher incidences and prevalences include communities with higher rates of intravenous drug use and prison populations [5]. Other risk factors include transfusions before 1992, intranasal cocaine use, infected donor organ transplantation, vertical transmission through infected mothers, hemodialysis, high-risk sexual activity, and high-risk occupations. Drug use is the most common in the United States, at 60% [6].

HCV is a Hepacivirus of the *Flaviviridae* family, with a positive-sense, single-stranded RNA genome that is post-translationally processed into structural and non-structural proteins, depending on host- and virally-encoded enzymes [7]. Although incompletely understood, the disruption of the innate antiviral interferon (IFN) pathway likely occurs via the inhibition of IFN-stimulated gene translation, localizing viral replication to cell compartments that are inaccessible to IFN-stimulated effector systems, or by the direct antagonization of IFN effector systems using viral proteins, which allows for infection persistence [8]. Eluding the adaptive immune response is most notable in chronic infection via the functional and phenotypical altering of Natural Killer cell and T cell responses that attempt unsuccessfully to clear the virus while nonetheless contributing to hepatocyte inflammation, with similar elevations in ALT and eventual fibrosis, as observed in HBV [8]. Antigenic variation, the rapid mutation of a genome through a lack of proofreading in the RNA-dependent RNA polymerase, allows an additional method of defense from the adaptive immune system by reducing antibody interaction and T cell recognition, consequently preventing the development of a reliable vaccine [9].

Fibrosis is a chronic process. Most hosts may be unaware of infection from a symptom perspective. Early courses of the disease, if symptomatic, will typically involve weakness, anorexia, right upper abdominal quadrant pain, dark colored urine, lower extremity edema, and jaundice. This usually occurs within seven to eight weeks. However, the majority will have either no or mild symptoms [10,11]. Approximately 30% of individuals will spontaneously clear the virus within six months. The remaining infected individuals often undergo long-term, low-grade inflammation that progresses to fibrosis and subsequently to cirrhosis in approximately 33% individuals, ultimately increasing the risk for HCC [12]. The rate of progression to fibrosis and cirrhosis is correlated with the degree of ALT elevation [13]. 

Hepatitis C is a multiorgan disease, as it causes both intra- and extrahepatic damage through immune complex deposition and direct cytotoxic effects. HCV-associated extrahepatic manifestations include cardiovascular disease, membranoproliferative glomerulonephritis, insulin resistance, lichen planus, keratoconjunctivitis sicca, vasculitis, cryoglobulinemia, lymphoproliferative disorders such as lymphoma, psychiatric disorders, and polyarthritis [14,15].

These extrahepatic manifestations lead to significant morbidity and mortality. However, it is difficult to separate Hepatitis C-related mortality due to the virus’s effect on these common disease processes. 

### 1.3. Hepatitis B

The Hepatitis B virus (HBV), known previously as “serum Hepatitis” after a smallpox-vaccine-related outbreak, underwent numerous additional outbreaks until the 1966 discovery of the “Australia antigen”, known today as HBsAg. Since that time, the World Health Organization (WHO) estimates that 296–364 million people have harbored a chronic HBV infection, or 3.8–4.9% of the world’s population, with 820,000 deaths occurring due to HBV-related causes [1,16]. The number of deaths is likely much higher in reality, as the collection of accurate data is limited by funding and technological availability [17].

Geographically, HBV infection and the lack of immunization predominates in the Western Pacific and African regions, with ethnic and racial disparities existing amongst non-U.S.-born persons and Asian communities, both foreign- and U.S.-born [17,18]. Transmissions occur most often via vertical transmission, namely via transplacental or perinatal infection from mother to infant, accounting for 42.1% of unvaccinated- and 2.9% of vaccinated-infant infections [19,20]. Other infection routes include sexual contact (which predominates in low endemic areas), illicit drug use, infected donor organ transplants, and other unsafe injections, including blood transfusions, dialysis, and other contaminated medical instruments, particularly in areas with poor screening [20].

HBV is an Orthohepadnavirus of the family *Hepadnaviridae*, containing a circular, partially double-stranded DNA genome that becomes fully double-stranded inside the host nucleus. HBV integrates into host cell genomes using reverse transcriptase. It then reduces the pathogen-associated molecular patterns in its genome and drives structural changes to enhance its resemblance to host cellular mRNA, concealing itself from innate immune system detection [21]. Exhaustive binding and direct protein inhibition further impair its recognition by the innate immune system. The adaptive immune response is impaired by NK cell phenotypic altering and the selective loss of the highly immunogenic HBeAg [21,22].

The virus itself is not cytopathic. However, attempts at viral eradication by the immune system result in damage to the host cell via the activation of CD4 T helper cells, CD8 T cells, NK cells, and inflammatory cytokines. The hepatocytes under this stress undergo fibrotic changes in response.

Although many patients may present with an asymptomatic disease, others will have nonspecific symptoms, including anorexia, nausea, abdominal pain, dark urine, clay-colored stool, and jaundice [23]. Spontaneous infection clearance occurs in 95% of those infected in adulthood, while the remaining 5%, in addition to the 90% of those with a vertically transmitted infection, develop a chronic disease that may advance to cirrhosis, liver failure, and hepatocellular carcinoma (HCC) [24]. Ascites, or bleeding due to the sequelae of portal hypertension, coagulopathies, encephalopathy, and a propensity for infection, are the evidence of a significant hepatic disease. Despite the lack of direct cytotoxic effects, indirect extrahepatic manifestations via immune complex deposition may result in complications such as a serum sickness-like syndrome, glomerulonephritis, polyarthritis, polyarteritis nodosa, cryoglobulinemia, Guillain-Barré syndrome, and various dermatoses [25].

## 2. Methods

We conducted a search through the National Center of Biotechnology Information’s (NCBI) PubMed database, Google Scholar, and Elsevier Clinical Key using a combination of the following keywords: Hepatitis C, Hepatitis B, Viral Hepatitis, Cirrhosis, Liver disease, Chronic, Fibrosis, Regression, Reduction, Mortality, Extrahepatic Manifestations, and Death. We limited our review from 1986 to the present. We used only publications written in English. These studies were evaluated prior to their inclusion in our review. References found within articles to key premises were also reviewed for inclusion. Our review did not contain strict inclusion or exclusion criteria. The intent was to identify articles that explicitly examined liver fibrosis- and Hepatitis-related deaths. We chose to exclude articles that focused on healthcare costs and the morbidity of the diseases, although morbidity is related to mortality.

## 3. Regression of Fibrosis

### 3.1. Hepatitis C

Inflammation is a protective mechanism that the host’s immune system uses to combat invasive bacterial and viral pathogens. However, the chronic inflammation of hepatocytes is the hallmark of Hepatitis C virus (HCV) infection, resulting in severe liver damage and an eventual progression to liver fibrosis and cirrhosis [26]. Chronic HCV proteins directly modulate cellular signaling pathways and indirectly generate host antiviral immune responses, resulting in chronic inflammation. Chronic HCV infection is associated with an increased expression of pro-inflammatory cytokines and a variety of immune cells, including Kupffer cells [27]. HCV inflammasome formation is related to viral exposure to macrophages [28]. This multiprotein complex can sense viral pathogen-associated molecular patterns (PAMPs) through their NOD-like receptors (NLRs), resulting in an increased expression of the cytokines IL-1B and IL-18 [29]. 

Liver fibrosis is identified by the accumulation of extracellular matrix (ECM) proteins, and is a byproduct of a persistent inflammatory response [30]. The hepatic stellate cell (HSC), which resides in the perisinusoidal space of Disse, is key in the generation and progression of liver fibrosis. HSCs are typically quiescent but mount a robust cellular response in the presence of inflammatory stimuli by transforming into proliferative myofibroblasts. HSC activation consists of three phases: initiation, perpetuation, and final resolution. The perpetuation phase involves autocrine and paracrine loops and is responsible for inducing liver fibrosis [31]. These activated HSCs rapidly contribute to ECM protein production, resulting in a predominance of ECM protein deposition over dissolution. In the setting of a sustained inflammatory stimulus, such as a chronic HCV infection, the chronic disruption of ECM protein equilibrium eventually leads to fibrotic scarring [32]. Experimental data have proposed that non-structural HCV proteins and HCV core proteins play a direct role in HSC activation via pro-mitogenic intracellular pathways and pro-inflammatory pathways, which include NF-kβ and JNK [33]. These proteins have also been shown to upregulate NADPH oxidase within hepatocytes, resulting in increased oxidative stress, and to modulate inflammatory pathways within monocytes via Toll-like receptor 2 (TLR2), resulting in hampered innate immunity [34,35]. The variable expression of MicroRNA has also been observed to play a role in stimulating HSC activity in individuals with chronic HCV. These MicroRNAs are transported from infected hepatocytes to HSCs. The downregulation of let-7 has been associated with the activation of HSCs via the targeting of TGF-β pathways. Other MicroRNAs, including MicroRNA-27a and MicroRNA 27b, have been shown to target specific receptors, such as retinoid X receptor α, which are crucial to the inhibition of HSC proliferation. The HCV-associated downregulation of MicroRNA-449a and MicroRNA-107 have further been shown to be associated with an increased expression of major inflammatory cytokines and STAT3, which contribute to the development of hepatic fibrosis [26].

Addressing liver fibrosis progression in patients with chronic HCV is paramount, as 20–30% of patients who do not receive treatment will develop cirrhosis over a 25–30-year period [12]. Historically, it was believed that liver fibrosis was irreversible, but with the development of novel direct antiviral therapies, fibrosis is now thought to be a dynamic process, with data suggesting the possibility of fibrosis regression in the setting of a sustained virological response.

The definition of liver fibrosis regression has not been formally established in the current medical literature. Although clinical observations of the current data show a reduction in the fibrosis content of the liver, they do not consider other potential markers of fibrosis regression, including changes in hepatic nodule size, hepatocyte regeneration, the extent of venular collapse, or alterations in the distribution of ECM proteins.

A variety of mechanisms at the cellular level have been postulated to explain the process of fibrosis regression. This has primarily been carried out in animal models. The three established cellular responses by HSCs to fibrosis regression are as follows: a return to a state of inactivity (quiescent state), apoptosis/autophagy, and cellular senescence [36]. Interestingly, HSCs that have returned from an activated to a quiescent state are thought to be more sensitive to future stimuli from inflammation and might be responsible for an accelerated progression of liver fibrosis [37]. Fibrous septa resorption, apoptosis and senescence of active HSCs, ECM degradation, collagenase enzyme stimulation and myofibroblast deactivation have all been observed to occur during fibrosis regression [38].

Although the probability of fibrosis regression is greater than cirrhosis remission, there are various factors, such as age, BMI, genetics, the rate of fibrosis progression, and the staging of chronic liver disease, that play a role in the amount of anticipated fibrosis regression and the rate at which fibrosis regression occurs. Moreover, the overlap of exogenous hepatotoxic compounds, such as concurrent alcohol use, or other chronic medical conditions, such as liver steatosis from metabolic dysfunction-associated steatotic liver disease (MASLD) or diabetes, can interfere with fibrosis regression or even contribute to fibrosis progression, despite the patient achieving SVR [39]. In this context, it is crucial to recognize that there is an undefined window of time during which therapeutic intervention must occur to achieve liver fibrosis regression. If liver damage progresses beyond a certain critical threshold—which has not yet been clearly defined—achieving a sustained virologic response (SVR) will not result in fibrosis regression.

Experts in the field believe that significant regression is unlikely once severe architectural distortion, vascular collapse, and portal hypertension have occurred. This may be due to the expansive structural cross-linking of collagen [40]. Consequently, there is limited evidence that large regions of parenchymal destruction can be repopulated by regenerated hepatocytes. Additionally, vascular lesions in liver cirrhosis often persist, with modest signs of recovery to normal circulation in cirrhotic livers [41].

Currently, there are several scoring systems used in clinical practice to assist in diagnosing and staging liver fibrosis, such as Ishak, Knodell and METAVIR [42]. Historically, liver biopsy was considered the gold standard for fibrosis staging. However, this technique has its own set of limitations.

The analysis of a small tissue specimen may underestimate the extent of fibrosis in the liver as the progression and the severity of fibrosis is not uniform. The accuracy of the results are dependent on the expertise of the pathologists. Lastly, the procedure is costly and invasive.

The non-invasive tests available include direct and indirect serum biomarkers, Egy-Score, transient hepatic elastography, and extracellular vesicles such as pentraxin 3. In more recent studies, fibrosis regression following SVR with direct acting antiviral (DAA) therapy has not been monitored by traditional methods using histological scoring systems as liver biopsies are infrequently performed. The popularity of non-invasive liver fibrosis staging methods has resulted in current research using a paired or bi-paired, non-invasive technique to evaluate for the evidence of fibrosis regression [43]. Liver stiffness measurement (LSM) has been a particularly popular non-invasive tool in the assessment of liver fibrosis, given its ability to measure the degrees of fibrosis and its high diagnostic accuracy for F2–F4 [44,45].

**Figure 1:**
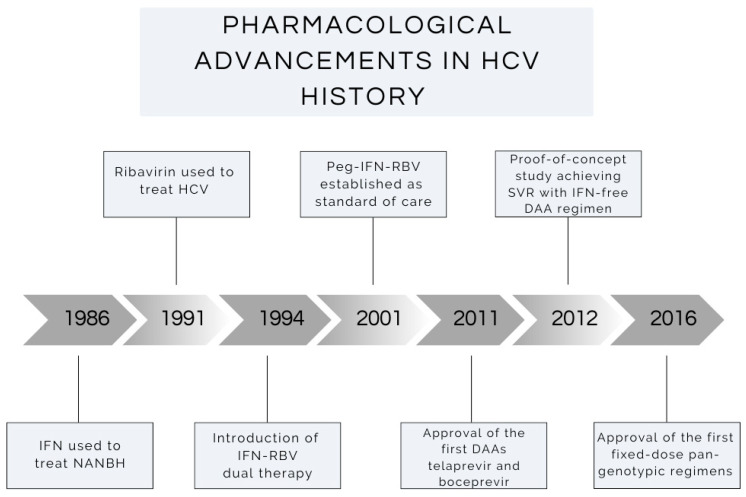
Graphical representation of the development of antiviral therapy for the treatment of Hepatitis C since 1986 [50,51,52,53,54,55,56,57].

Figure 1 displays a discovery timeline of antiviral therapy used to treat HCV. Table 1 outlines the studies conducted over the past 25 years exploring fibrosis regression in patients with HCV after treatment with IFN-based therapies and directacting antivirals. The evidence presented supports the thesis that patients with chronic HCV infection who achieve SVR experience significant reductions in liver fibrosis, irrespective of what DAA is used or the duration of the treatment. However, it is important to note that fibrosis regression following SVR is not universal.

A subset of patients continues to exhibit the progression of liver fibrosis, despite achieving SVR [46,47,48,49]. This is particularly evident in patients with coexisting conditions that predispose them to ongoing systemic inflammation and liver damage, as mentioned previously.

Moreover, the presence of hepatocellular carcinoma (HCC) at the time of SVR or the development of HCC after SVR further complicates the clinical course. Its presence signifies a more severe underlying liver disease that may not fully benefit from the antifibrotic effects associated with viral eradication. Patients with HCC may continue to experience liver fibrosis progression due to the tumor’s impact on the hepatic architecture and function.

**Table 1:** Fibrosis regression after treatment of HCV with IFN-based therapy and/or DAAs.

Study	N	Therapy	Follow up	Fibrosis Assessment Modality	Findings
Shiratori et al., 2000 [58]	487	IFN-based	1–10 years, Median of 3.7 years	Histology (METAVIR)	Mean reduction in fibrosis score of −0.60 +/−0.07 at <3 yr follow up and −0.88+/−0.08 at 3 yr or more follow up.
Poynard et al., 2002 [59]	1030	IFN-based	20 month mean duration between biopsies	Histology (METAVIR)	Reversal and/or regression of cirrhosis occurred in 49% of patients, where 15% regressed to stage III, 16% reversed to stage II, 15% reversed to stage I, and 2% reversed to stage 0.
Maylin et al., 2008 [60]	126	IFN-based	3.27 years	Histology (METAVIR)	Fibrosis stage improved in 56% of patients. Cirrhosis regression was observed in 64% of patients.
D’Ambrosio et al., 2012 [61]	38	IFN-based	61 months	Histology (METAVIR)	Reversal of cirrhosis occurred in 23.6% of patients, regression of cirrhosis in 61%, and regression of fibrosis in 36%.
Knop et al., 2016 [62]	54	DAA	24 weeks	TE, ARFI	At 24 weeks, 88% showed an improvement in LS values, and 46% showed a reduction in liver stiffness by >30%.
Bachofner et al., 2016 [63]	392	DAA	18 months	TE, FIB-4	Overall TE values decreased, with a regression of 32.4%. Median FIB-4 values decreased from 2.54 to 1.10.
Dolmazashvili et al., 2017 [64]	304	IFN/DAA	24 weeks	TE	Of the patients, 65.1% achieved a reduction in LS by at least 20%. Percentage of patients with stage F4 decreased from 56.6% to 36.5%.
Facciorusso et al., 2017 [65]	153	INF-based	6 months, then every year for 5 years	TE	LS at baseline was 12.8 kPa (9.3–18.7). At EOT, median LS decreased to 10.7 (8.5–16.2), while at SVR24, it reached 9.6 (7.9–14.3). LS resulted 8.7 kPa (7.1–13.7) at 1 year, 8.23 (6.8–12.5) at 2 years, and 7.9 (6.2–10.8) at 3 years, whereas LSM was 7.7 (6.2–9.2) and 7.3 (5.9–8.3) at 4 and 5 years, respectively.
Pietsch et al., 2018 [46]	143	DAA	24 and 96 weeks	TE, FIB-4 and APRI	LS reduction was seen in almost every patient, irrespective of liver disease stage. Further regression seen at 96 weeks in patients with early cirrhosis, not those with cirrhosis and impaired liver function. Progression of liver stiffness progressed, despite SVR in 17% of patients.
Hedenstierna et al., 2018 [39]	269	DAA	7.7 years	TE	Majority of patients improved their fibrosis stage after SVR. Of the patients, 24% had persistent advanced fibrosis with a LS > 9.5 kPa.
Shiha et al., 2020 [47]	2326	DAA	12–45 months	TE, FIB-4, FIB-5 and APRI	Cirrhotic patients: 21.8% showed reversal of cirrhosis, 27.4% showed fibrosis regression, and 50.8% remained unchanged. Of the F3 patients, 26.5% showed reversal of fibrosis, 31.5% showed fibrosis regression, 30.6% remained unchanged, and 11.4% progressed to F4.
Hablass et al., 2021 [66]	137	DAA	12 months	TE and FIB-4	FIB-4 and TE values after HCV elimination were significantly lower than their mean values in all patients.
El-Kady et al., 2021 [67]	300	DAA	2 years	TE	Both DAA treatment regimens showed improvement in liver fibrosis.
Zakareya et al., 2021 [68]	655	DAA	1, 3 and 5 years	TE	Overall regression of liver stiffness in all patients with SVR.
Agwa et al., 2022 [69]	1230	DAA	12 and 48 weeks	TE, FIB-4	Of the F4 patients, 42.7% improved to F3-F1. Of the F3 patients, 66% improved to F2-F1. Of the patients, 28.4% improved from significant fibrosis (≥F3) to non-significant fibrosis (≤F2).
Hassanin et al., 2022 [70]	162	DAA	48 weeks	TE, FIB-4 and TIMP-1	Liver fibrosis improved in all patients, with significant declines in TIMP-1 levels, LSM, and FIB-4 score.
Thanapirom et al., 2022 [71]	89	DAA	1 year	TE, SWE, and MRE	Viral eradication resulted in the reduction of LS values.
Chuaypen et al., 2022 [72]	83	DAA	12, 24 and 72 weeks	MRE	Patients who achieved SVR showed mean MRE decreased significantly from baseline to FUw24 and FUw72. At FUw72, patients with baseline F2-F4 had a higher rate of ≥30% MRE decline compared with individuals with baseline F0-F1.
Rosato et al., 2022 [48]	516	DAA	24 months	TE	Significant decrease in LS until SVR, with a progressive reduction until 24 months. Patients with steatosis and those who developed HCC did not show a late improvement in LS.
Yoo et al., 2022 [73]	112	DAA	48, 96 and 144 weeks	TE, FIB-4 and APRI	In patients who achieved SVR, LS values significantly decreased over time (19.4 ± 12.9 kPa [baseline], 13.9 ± 9.1 kPa [48 wk], 11.7 ± 8.2 kPa [96 wk], 10.09 ± 6.23 [144 wk]).
Niu et al., 2023 [74]	347	DAA	12 weeks	TE, FIB-4, APRI and GPR	For the elderly group, the median LSM was 11.6 (7.9–19.9) kPa, and this value was significantly reduced to 9.7 (6.2–16.6) kPa. Similarly, GPR, FIB-4, and APRI indices were significantly reduced from 0.445, 3.072, and 0.833 to 0.231, 2.100, and 0.336.
Abu-Freha et al., 2023 [49]	209	DAA	≥3 years	VCTE	Fibrosis improvement was observed among 57% of patients; fibrosis progression was seen among 7% of patients; and no change was seen in 36% of patients. Of these patients, 28% regressed from F3/F4 to F2 or less.
Singh et al., 2017 [75]	24 studies	DAA or IFN	VCTE prior to therapy and at least 1 follow up VCTE	VCTE	Eradicaiton of Hepatitis C is associated with significant decrease in liver stiffeness, especially in patients with high levels of inflammation, or if the patient was treated with DAA therapy.

HCC: hepatocellular carcinoma; DAA: direct acting antiviral; IFN: interferon; SVR: sustained virological response; SWE: shear wave elastography; MRE: magnetic resonance elastography; TE: transient elastography; ARFI: Acoustic Radiation Force Impulse; LSM: liver stiffness measurement; TIMP-1: tissue inhibitor of metalloproteinases-1; GPR: gamma-glutamyl transpeptidase to platelet ratio; VCTE: vibration controlled transient elastography.

### 3.2. Hepatitis B

The progression of liver fibrosis in chronic HBV patients is facilitated by a combination of extracellular factors, growth factors, and the upregulation of pro-inflammatory gene expression. HBV and its related surface protein have been shown to directly activate HSCs and influence collagen protein production via the transient infection of the HSC and the upregulation of collagen type I mRNA [76]. Further paracrine activation occurs by the infected hepatic cell’s secretion and the transport of HBV-encoded oncogene X protein (HBx) and other MiRNAs into the HSCs. The Wnt pathway, HMGB1, monocytes, Th17 cells, and NK cells have also been implicated in HSC activation [77]. TLR4, FAP, a-SMA, TIMP1, and COL1A1 have all been shown to be upregulated in patients with chronic HBV and advanced fibrosis. Cytokines, such as IL-1β and TNF-α, were found to be upregulated in these patients, which correlates with a more profound inflammatory response, suggesting they may play a pivotal role in fibrosis progression [78]. IL-21 is a cytokine that is activated by T cells and plays a crucial role in the regulation and survival of CD4+ and CD8+ cells. The modulation of IL-21 production in chronic HBV leads to an impaired immune response against cells infected with HBV, resulting in the compromised non-cytolytic control of Hepatitis B replication [79]. This is an important factor as the progression of liver fibrosis has been observed to occur in some patients with chronic HBV, despite antiviral therapy, due to persistent low levels of HBV [80]. The most serious long-term consequence of persistent HBV infection and progression of hepatic fibrosis is the development of HCC. Chan et al. conducted a retrospective cohort study that showed metabolic dysfunction-associated fatty liver disease (MAFLD) was an independent risk factor for the development of HCC in patients with chronic HBV that was associated with the presence of the *APOC3* gene polymorphism [81]. This gene is expressed in the liver and plays an integral role in fatty acid metabolism. This is critical, given the ramifications regarding increased morbidity and mortality with HCC.

The concept that treating Hepatitis B in the setting of liver fibrosis can result in the regression of fibrosis being established. The first trial that supported this hypothesis was conducted in 1999, in which chronic Hepatitis B patients who received antiviral therapy were noted to have a regression of their liver fibrosis [82]. Since then, multiple trials using different antivirals have demonstrated similar results, as demonstrated in Table 2.

Interferon Alfa 1990, lamivudine 1998, Adefovir 2002, Telbivudine 2006, Entecavir 2005, and Tenofovir Disoproxil Fumarate 2008 were FDA-approved drugs for treating chronic Hepatitis B, in such order [83]. Head-to-head trials were done, comparing the efficacy of treatment as well as fibrosis regression. Originally, Lamivudine was used for chronic Hepatitis B treatment. Lamivudine achieved adequate viral replication suppression and reduced serum HBV DNA; however, emergence of lamivudine resistant mutants (tyrosine–methionine–aspartate–aspartate (YMDDs)). The frequency of this HBV polymerase gene mutation increases with the duration of therapy [84]. Adding Adefovir to combat Hepatitis B lamivudine resistance was approved in 2002. This resulted in a significant improvement in the treatment, with higher ALT normalization, as well lower serum HBV DNA levels [85]. Both Entecavir and Tenofovir were approved later for Hepatitis B treatment and showed superiority to Adefovir.

Currently, Entecavir and Tenofovir are the first line recommended antiviral therapies for people with chronic HBV infection [86]. A randomized, open-label study demonstrated that Entecavir showed superiority to adefovir in achieving a reduction in serum Hepatitis B virus, as well lower HCC [87]. Another trial that compared Tenofovir Disoproxil Fumarate (TDF) to adefovir demonstrated that tenofovir resulted in a lower viral load and the normalization of ALT [88].

Several factors, such as viral load, ALT level, HBeAg, and HBsAg, are monitored during treatment for liver fibrosis. A recent study done by Oh et al., 2023, demonstrated that an improvement in the FIB-4 index and an undetectable HBV DNA level at one year are associated with a significantly lower risk of HCC [89]. Patients who do not achieve CR compared to those who do are at a higher risk of HCC [89,90].

Scoring fibrosis is important to study the disease process. The Ishak fibrosis score is frequently used in clinical trials to classify liver biopsy histology. The P-I-R classification, which was originally referred to as the Beijing Classification, was introduced in 2017 for the evaluation of liver fibrosis regression in chronic Hepatitis B patients after antiviral therapy [91]. The objective of the system is to classify patients into one of three categories: progressive, indeterminant, and regressive.

A randomized clinical trial of 646 Hepatitis B patients on entecavir-based therapy for a duration of seven years compared the utility of the P-I-R classification to the Ishak score in identifying clinical outcomes [91]. The P-I-R classification was able to better predict clinical outcomes as well as further differentiate the histological outcomes in patients with stable Ishak scores. An advantage of the P-I-R system is that it tends to be more binary between worsening or improving (progressive vs. regressive) compared to Ishak scoring. Of the patients, 218 were identified with a stable Ishak score, but only 44 of those patients fell into the P-I-R indeterminant category. Most of the patients who developed HCC from the stable Ishak group were identified as progressive on the P-I-R scoring system, which could mean that the P-I-R classification could better differentiate the HCC risk [91].

The duration of therapy has been shown to have an inverse linear relationship with the regression of fibrosis. A randomized clinical study that included 641 Hepatitis B patients on Tenofovir (TDF) had a significant finding of a decreased Knodell score as well as a decreased Ishak score when comparing patients’ liver biopsies from the baseline at 1 year and at 5 years [92]. Patient’s 5-year biopsies had lower Knodell scores and Ishak scores when compared to patient’s 1-year biopsies, which indicates that a longer duration of treatment might result in better outcomes.

**Table 2:** Fibrosis regression after treatment of HBV with DAAs.

Study	N	Therapy	Follow up	Findings
Suzuki et al., 1999 [82]	24	Lamivudine	52 weeks	Of the patients, 95% had a reduction of their hepatic HAI necroinflammatory score by ≥2 points.
Dienstag et al., 2003 [93]	63	Lamivudine	36 weeks	Of the patients, 57% showed ≥2 points reduction in the HAI necroinflammatory score, 33% showed no significant changes, and 11% showed worsening. Of the patients who had bridging fibrosis (HAI fibrosis score of 3) at the start of study, 63% had ≥2 point reduction in the HAI fibrosis score. Patients with YMDD variants were least likely to improve.
Hadziyannis et al., 2006 [94]	185	Adefovir	240 weeks	Of the patients, 73% and 83% had improvement of fibrosis and necroinflammation after receiving adefovir for 192 and 240 weeks, respectively. Continued-Adefovir group had a reduction of 4.7 points from baseline in the overall HAI at week 96. Continued-Adefovir group had significant reduction from baseline in the Ishak fibrosis score at week 96.
Yokosuka et al., 2009 [95]	167	Entecavir	120–146 weeks	Twenty-one patients had paired baseline and on treatment biopsies. Of the patients, 100% (21/21) had ≥2 point decrease in HAI necroinflammatory score. Of the patients, 57% (12/21) had ≥1 point decrease in HAI fibrosis score.
Schiff et al., 2008 [96]	1633	Entecavir and Lamivudine	48 weeks	Entecavir: Of the patients, 80% who were HbeAg positive, 75% who were HBeAg negative, and 57% who were lamivudine refractory HBeAG negative had ≥2 point decrease in HAI necroinflammatory score. Of the patients, 57% who were HBeAg positive, 59% who were HBeAg negative, and 43% who were lamivudine refractory HBeAg negative had ≥1 point decrease in Ishak score. Lamivudine: Of the patients, 64% who were HbeAg positive, 60% who were HBeAg negative, and 29% who were lamivudine refractory HBeAG negative had ≥2 point decrease in HAI necroinflammatory score. Of the patients, 49% who were HBeAg positive, 53% who were HBeAg negative, and 33% who were lamivudine refractory HBeAg negative had ≥1 point decrease in Ishak score.
Marcellin et al., 2013 [92]	641	Tenofovir DF	240 weeks	The proportion of patients with mild or no necroinflammation (Knodell HAI score 0–3) increased from 8% at baseline to 80% at year 5, indicating ≥ 2 point reduction in HAI necroinflammatory score. The proportion of patients with no or mild fibrosis (Ishak score 0–2) increased from 39% at baseline to 63% at year 5, indicating ≥1 point reduction in Ishak score. Of the patients with cirrhosis at baseline, 58% had ≥3 point decrease in Ishak score at year 5.

HBeAg: Hepatitis B e-Antigen; DF: disoproxil fumarate; disease progression: hepatic decompensation, HCC, SBP, bleeding varices, death related to liver disease; HAI: histology activity index.

## 4. Effect on Mortality

### 4.1. Hepatitis C

Hepatitis C (HCV) differs from HBV in two important ways from a mortality perspective. First, HCV is a curable viral infection. Second, extrahepatic manifestations (EHMs) may be less pronounced when compared to glomerulonephritis and polyarteritis nodosa, a necrotizing vasculitis, seen in HBV, but still contribute substantially to mortality. Unlike fibrosis, which is measurable with direct or indirect testing, mortality that is clearly linked to cirrhosis or extrahepatic manifestations is more difficult to quantify. For example, cardiovascular disease alone is a significant driver of mortality worldwide, with risk factors that are independent of Hepatitis C. Table 3 summarizes key studies relevant to this discussion.

Establishing a baseline mortality rate to demonstrate improvement with antiviral therapy remains difficult. Most recently, a large retrospective cohort using Taiwanese population data found the mortality rate in an untreated population to be 0.24% per person–month [97]. In England, the all-cause mortality of HCV-infected individuals per person–year is 2.3 times higher than that of the general population. This number increases to 4.7 if the individual is aged 30–69 yrs old [98]. This study did not distinguish treated from untreated individuals. From the US Department of Veterans affairs from 2001 to 2008, patients with genotype 1–3 Hepatitis C were given pegylated interferon and ribavirin. Patients who achieved sustained virologic response (SVR) had a 30–49% mortality risk reduction [99]. A significant mortality reduction is maintained if SVR is achieved using DAA [100].

**Figure 2:**
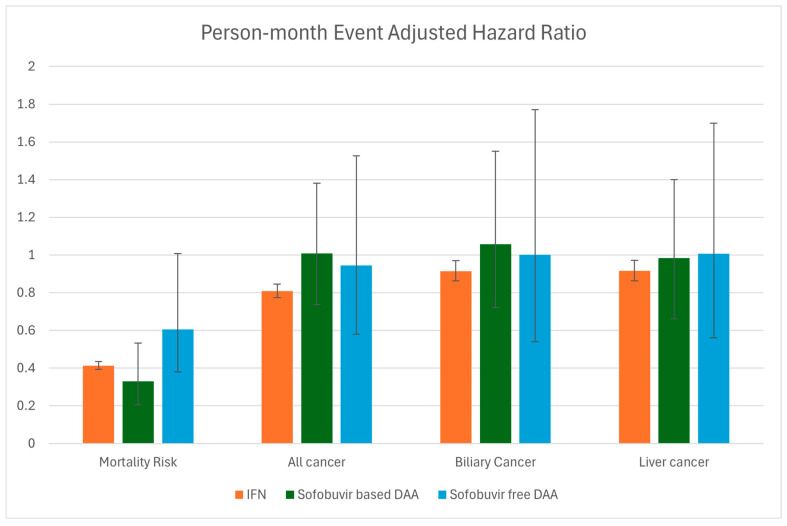
Data adapted from Chen et al., 2024 [97]; Event Adjusted Hazard ratio separated by therapy type. No cancer had a statistically significant reduction with DAA therapy, as demonstrated by crossing the significance threshold of 1.0. IFN-based therapy reduced the risk of mortality, all cancers, biliary cancer, and liver cancer.

With today’s direct-acting antiviral therapies, a virologic cure is achievable in nearly all patients with Hepatitis C. This significantly reduces mortality from the sequelae of portal hypertension, and all-cause mortality [100,101]. However, the HCC risk is less clear. Once data was adjusted for variables, the HCC risk was decreased by 34% as a result of DAA therapy [100]. These findings were not replicated by Chen et al., 2024. All-cause mortality did decrease in both the DAA groups and the IFN group. HCC and extrahepatic malignancies, such as non-Hodgkin lymphoma, were only reduced in the IFN group. This is represented graphically in Figure 2. All patients with chronic Hepatitis C should be treated with antiviral therapy but may need increased cancer surveillance for extrahepatic solid tumors and hematologic malignancies.

Extrahepatic manifestations such as cardiovascular disease, insulin insensitivity, chronic kidney disease, stroke, and mental disorders cause significant mortality in patients with chronic HCV infection. Treatment with direct-acting antivirals significantly reduces the risk of death from these disease processes by as much as 75% per 1000 person–years [102].

Moreover, recent studies have demonstrated that achieving SVR can not only reduce the risk of death from these diseases that are already present among patients but also reduce the incidence. A prospective study by Adinolfi LE et al. demonstrated that achieving SVR resulted in an 81% risk reduction in developing Type II Diabetes Mellitus in patients with both mild and advanced fibrosis [103].

It is known that chronic HCV increases the risk for both subclinical and clinical cardiovascular disease via direct and indirect pro-atherogenic mechanisms, including immune responses, metabolic disturbances, and a direct cardiovascular tropism of the virus [104]. A recent multicenter prospective study showed that achieving SVR through DAA treatment significantly reduced the risk of major cardiovascular events in patients, irrespective of the degree of hepatic fibrosis across all age groups studied [105].

The incidence of non-liver malignancies have been noted to be lower in those treated with DAAs compared to untreated individuals when adjusted for potential confounding factors such as age, sex, and ethnicity [106].

Neurologic and psychiatric disorders are reported in half of patients with chronic Hepatitis C. As the virus can replicate within the brain, there may be some direct neurotoxicity caused by viral infection. This can manifest neurologically as myelitis, encephalopathy, and encephalomyelitis. The psychiatric manifestations are depression, anxiety, fatigue, and persistent neurocognitive impairment (forgetfulness, loss of concentration, and sluggish thinking). An overview published in 2015 by Adinolfi et al. specifically examined this topic. However, it was limited to IFN therapy. More specific studies need to be conducted to understand the reversibility and impact of therapy [107].

However, curing the infection does not necessarily lead to a mortality rate equal to the general population [108]. This study was limited as it did not include alcohol, drug use, and the possible sequelae of EHMs. It does, however, highlight the need for long-term medical follow up for patients treated for HCV.

The degree of existing liver injury does influence the mortality reduction associated with antiviral therapy. The findings of a large French cohort study suggest that baseline cirrhosis and exposure to direct-acting antivirals was strongly associated with decreased all-cause mortality. However, this study was limited as fibrosis was not assessed during treatment nor follow up and was only checked upon entry into the cohort [100]. Further study would be needed to address the degree of mortality reduction relative to liver fibrosis level at the time of antiviral therapy initiation.

**Table 3:** Effect of antiviral therapy on Hepatitis C mortality and extrahepatic manifestations.

Publication	Description	N	Intervention/Populations	Endpoints	Follow-Up	Conclusion
Backus et al., 2011 [99]	VA observational study of Hepatitis C patients without HIV, treated with pegylated interferon and ribavirin, examining all-cause mortality in patients with sustained virologic response to therapy.	16,864	Pegylated interferon and ribavirin	All-cause mortality.	6 yrs	Pegylated interferon and ribavirin therapy that achieved SVR was associated with a significant reduction in all-cause mortality.
Butt et al., 2019 [109]	VA observational study of Hepatitis C patients without HIV or HBV, treated with pegylated interferon or direct-acting antivirals, examining treatment effect on cardiovascular disease events.	17,103	Pegylated interferon and ribavirin or direct-acting antivirals	Cardiovascular disease events > 12 weeks after treatment initiation for Hepatitis C, based on 1 inpatient or 2 outpatient ICD-9 or 10 codes for myocardial infarction, unstable angina, congestive heart failure, percutaneous transluminal coronary angioplasty, coronary artery bypass grafting, and stroke.	10 yrs	Direct-acting antiviral therapy was associated with a 43% reduction, and PEG/interferon was associated with a 22% reduction in CVD events.
Cabibbo et al., 2019 [110]	Prospective cohort study examining the effect of direct-acting antiviral medications on the overall survival of patients with compensated cirrhosis and HCC, post resection or ablation.	491	Direct-acting antivirals vs. none	Overall survival from therapy to death or last visit for both DAA vs. no-DAA.	Mean: 21.4 months	DAA improved overall survival and reduced the risk of hepatic decompensation; however, risk of HCC recurrence was not significantly reduced.
Carrat et al., 2019 [100]	Prospective study examining all-cause mortality, HCC, and decompensated cirrhosis in patients treated with direct-acting antivirals, compared untreated	10,166	Treating Hepatitis C with direct-acting antivirals	All-cause mortality, HCC, and decompensated cirrhosis.	33.4 months	Treatment with DAA was associated with reduced risk for mortality and HCC and should be considered in all patients with chronic Hepatitis C.
Chen et al., 2024 [97]	Retrospective cohort study comparing Hepatitis C morbidity and mortality of patients treated with DAA or INF vs. untreated patients.	117,450	INF or DAA vs. no treatment	Morbidity and mortality.	3-year intervals	Antiviral therapy inducing a sustained virologic response improves morbidity and mortality, but there are cancer rate differences in INF vs. DAA treatment modalities.
Gastaldi et al., 2019 [111]	Single-arm exploratory trial with 17 non-diabetic, lean chronic Hepatitis patients without significant fibrosis, treated with sofosbuvir/ledipasvir and ribavirin for 12 weeks. Insulin sensativity and cytokine levels were analyzed before and after treatment.	12	Sofosbuvir/ledipasvir and ribavirin	Insulin sensitivity and cytokine levels.	6–24 weeks after antiviral therapy	Inhibition of HCV improves peripheral insulin sensitivity in non-diabetic, lean individuals with chronic Hepatitis C without significant fibrosis.
Hamill et al. 2023 [108]	Population-based cohort study to quantify the mortality rates of patients successfully treated for Hepatitis C with DAA, compared to the general population.	21,790	None	Mortality rate.	12 weeks after antiviral therapy until death or 31 December 2019	Mortality rates of patients successfully treated for Hepatitis C remains high.
Ireland et al., 2019 [98]	Observational cohort study to estimate mortality rates of individuals diagnosed with Hepatitis C in England from 2008 to 2016.	43,895	None	Mortality rate.	2008–2016	Mortality rate of Hepatitis C patients is higher than the general population.
Janjua et al., 2021 [112]	Population-based cohort study examining effect of direct-acting antiviral-induced sustained virologic response on all-cause-, liver-, and drug-related mortality of chronic Hepatitis C patients.	10,851	Direct-acting antivirals vs. none	Death.	mean 2.2 years	DAA treatment is associated with a substantial reduction in all-cause-, liver-, and drug-related mortality
Jeong et al., 2024 [102]	Population-based cohort comparing extrahepatic manifestations-related deaths in patients with chronic Hepatitis C treated with DAA vs. untreated.	25,630	Direct-acting antivirals vs. none	Extrahepatic manifestation mortality.	median 3.4 years	Treatment of chronic Hepatitis C with DAA was associated with significant reductions in extrahepatic manifestation-related mortality.
Mandorfer et al., 2016 [101]	Retrospective study examining the effects of INF-free SVR on portal hypertension in patients with HVPG ≥ 6mmHg.	104	Hepatic venous pressure gradient and transient elastography or transient elastography alone	Hepatic venous pressure gradient following SVR.	12 weeks after antiviral therapy	SVR may reduce HVPG in all baseline HVPG groups, but the effect is decreased in patients with more advanced liver disease.
Ogawa et al., 2022 [106]	Retrospective cohort study examining insurance claim data to examine the incidence of HCC, relevant non-liver events, and overall mortality.	245,596	Treatment with DAA with IFN	Incidence of HCC, liver decompensation, relevant non-liver events, and overall mortality.	11 years	A significant reduction in mortality was seen in patients with and without cirrhosis. Both liver and non-liver events were decreased with DAA therapy.

### 4.2. Hepatitis B

Mortality associated with chronic Hepatitis B is due to the sequelae of decompensated cirrhosis or hepatocellular carcinoma (HCC). Hepatitis B is currently incurable. However, antiviral therapy is effective for preventing disease progression, decreasing the cancer risk, normalizing liver enzymes, and reducing mortality. Moreover, antiviral therapy is well tolerated, even in those with advanced decompensated cirrhosis [87]. Jang et al. in 2015 demonstrated that the prompt administration of antiviral therapy significantly modifies the natural history of decompensated cirrhosis and increases survival [113]. Furthermore, 60% of treated patients listed for transplant were either removed from the transplant list due to improvement or were able to delay liver transplant.

**Figure 3:**
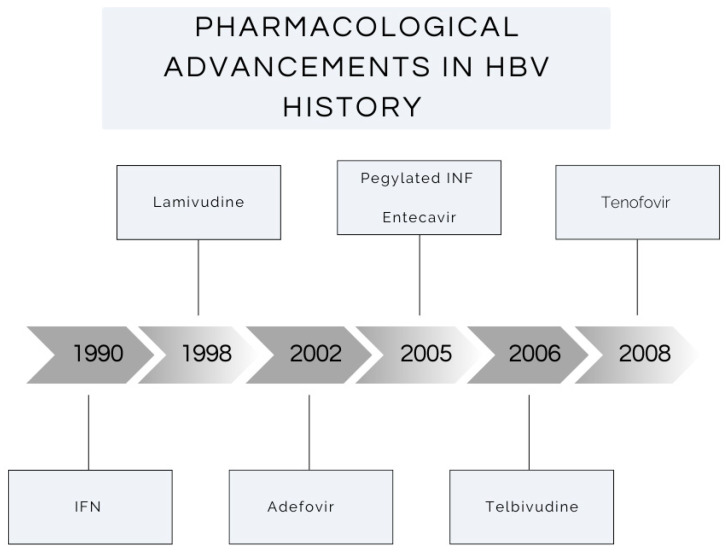
Graphical representation of the development of antiviral therapy for the treatment of Hepatitis B since 1990.

As above in Figure 3, antivirals have progressed over the last 34 years to include direct-acting antivirals, with the introduction of adefovir for use in Hepatitis B in 2002. The use of antivirals significantly reduces the HCC risk in those with Hepatitis B. Wu et al. conducted a retrospective, nationwide matched cohort study in 2014 in Tiawan of 43,190 patients, examining the incidence of HCC in two cohorts: patients receiving nucleoside analogue therapy and those who were untreated [114]. The treated cohort had a significantly lower 7-year incidence of HCC, at 7.32% compared with a 22.7% incidence in the untreated cohort.

Furthermore, DAA therapy is useful in patients who have already been diagnosed with HCC. Yuan et al. published a meta-analysis in 2016 examining the use of nucleosid(t)e analogues in patients who had undergone the curative resection of hepatocellular carcinoma. The use of these medications was associated with a significant reduction in HCC recurrence and increases in recurrence free survival at 1-, 3-, and 5-years post therapy [115].

Garg et al. conducted a double-blind, placebo-controlled trial in 2010, examining the use of tenofovir for the spontaneous reactivation of Hepatitis B presenting as acute-on-chronic liver failure. Survival at 90 days in the placebo group was 15%, versus 57% in the tenofovir group [116].

Extrahepatic malignancies contribute to the overall mortality of these patients. Chon et al. conducted a population-based, retrospective study using data from the Korean National Health Insurance Service and showed that among non-cirrhotic chronic Hepatitis B patients, extrahepatic malignancies were the leading cause of death, with a 10-year overall mortality rate of 0.23 per 100 person–years [117]. Hepatitis B patients are at an increased risk for developing non-Hodgkin B cell lymphoma [118,119]. Other extrahepatic malignancies associated with chronic Hepatitis B include colorectal, pancreatic, and gastric cancers [120,121].

Other extrahepatic manifestations include cryoglobulinemia, vasculitis, serum-sickness-like syndrome, non-rheumatoid arthritis, polyarteritis nodosa, membranous nephropathy, and membranoproliferative glomerulonephritis. This is important to recognize as roughly 20% of patients with chronic Hepatitis B will experience extrahepatic manifestations that directly influence the patient’s morbidity, quality of life, and mortality [122].

Table 4 outlines eight publications addressing the effects of antiviral therapy on Hepatitis B-associated mortality. These studies outline the significant progress towards mortality reduction, while noting the remaining room for continued advancement.

## 5. Conclusions

Direct-acting antiviral medications have been a pivotal advancement in medicine worldwide. These therapies are well tolerated, effective, and are becoming more widely available. This review highlights the significant potential for liver fibrosis regression in patients with Hepatitis B or Hepatitis C, particularly with the advent of direct-acting antivirals (DAAs). The studies examined demonstrate that achieving a sustained virologic response (SVR) in Hepatitis C patients leads to a marked decrease in liver fibrosis. This contributes to improved morbidity and mortality outcomes.

Similarly, antiviral treatments for Hepatitis B have shown promising results in slowing disease progression. These findings underscore the critical role of an early diagnosis and effective antiviral therapy in managing chronic viral Hepatitis and preventing long-term liver complications.

While this review provides a broad overview of Hepatitis B and Hepatitis C treatments, liver fibrosis regression, and mortality, several limitations should be acknowledged. The lack of strict inclusion and exclusion criteria in the literature selection process may have introduced a selection bias, as studies were chosen based on their explicit examination of liver fibrosis and mortality. This may have resulted in the exclusion of relevant studies that address these outcomes in a more nuanced or indirect manner.

We have elected to omit a discussion on immunosuppression-induced Hepatitis B reactivation. Although this can be a cause of morbidity and mortality, this is a nuanced topic that deserves substantial discussion that we have decided is tangential to the focus of fibrosis and mortality reduction.

Additionally, our search was limited to studies written in English, potentially skewing the data toward regions where English publications predominate. Moreover, the exclusion of studies focusing on healthcare costs and morbidity, although justified by our focus on mortality, may overlook important factors that contribute to patient outcomes and disease progression. Finally, we relied on electronic databases, which, although comprehensive, might not capture all the relevant gray literature or the non-indexed studies, potentially limiting the completeness of our findings.

Exploring the long-term impact of new antiviral therapies, such as direct-acting antivirals (DAAs) for Hepatitis C and advances in Hepatitis B treatments, could further inform future reviews on how these therapies contribute to reductions in both fibrosis progression and mortality. Furthermore, integrating studies that evaluate not just mortality but also healthcare cost and morbidity could provide a more holistic perspective on the overall burden of Hepatitis-related liver disease. As the landscape of antiviral therapy continues to evolve, continuous updates to the literature will be essential to capturing the latest advancements in treatment efficacy and outcomes.

There is still significant room for improvement regarding screening at-risk populations and improving access to these critical medications. Hopefully, the future will bring continued antiviral therapy advancement and a decrease in the healthcare burden caused by Hepatitis B and Hepatitis C.

## Figures and Tables

**Table 4:** Effect of antiviral therapy on Hepatitis B Mortality.

Publication	Description	N	Intervention	Endpoints	Follow-Up	Conclusion
Zhou et al., 2020 [123]	Population-based study examining liver-related and all-cause mortality in patients with chronic Hepatitis B, prior Hepatitis B infection, vs. no Hepatitis B infection.	39,206	None	All-cause mortality and liver-related mortality.	None. NHANES participants from 1999 to 2014	Mortality of patients with Hepatitis B infection still exceeds that of the uninfected patient, despite the use and availability of improved therapeutics.
Ge et al., 2020 [124]	Comparison of antiviral therapy in patients with chronic Hepatitis B and HCC who underwent curative resection	479	Tenofovir vs. other antiviral therapy	Overall survival and disease-free survival.	~80 months	Anti-virus treatment with TDF benefits for both overall survival and disease-free survival of CHB-related patients over other nucleos(t)ide analogues.
Yuan et al., 2016 [115]	Meta-analysis of 15 studies examining the impact on survival of antivirals after curative therapy in patients with HBV-related HCC.	8060	lamivudine, adefovir dipivoxil, and entecavir	Overall survival and recurrence-free survival.	Meta-Analysis	Analysis of the data from the 15 studies examined provided significant evidence of the benefits of using NAs, as the use of NAs was associated with a decreased HCC recurrence rate after curative therapy and improved RFS, especially long-term survival (more than one year).
Liaw et al., 2011 [125]	Double-blind study of 112 patients with chronic Hepatitis B with decompensated cirrhosis, establishing the safety of Tenofovir Desoproxil Fumarate, Emtricitabine/TDF, and Entecavir.	112	Tenofovir Desoprocil Fumarate, Emtricitabine/TFD, and Entecavir	Primary safety endpoints. Tolerability failure resulting in permanent treatment discontinuation, increase in serum creatinine > 0.5 mg/dL, or serum phosphorus < 2.	48 weeks	All treatments were well tolerated in patients with decompensated liver disease due to Hepatitis B.
Garg et al., 2010 [116]	Use of Tenofovir for spontaneous reactivation of chronic Hepatitis B causing acute or chronic liver failure.	27	Tenofovir vs. placebo	Survival at three months.	90 days	Tenofovir significantly reduces mortality vs. placebo.
Jang et al., 2015 [113]	Cohort study comparing the effect of antivirals on 5-year transplant-free survival of patients presenting with first-onset decompensation complications associated with Hepatitis B.	707	Antiviral therapy vs. none	Liver transplant-free survival.	5 years	Antiviral therapy significantly modifies the natural history of decompensated cirrhosis, improving liver function and increasing survival.
Wu et al., 2014 [114]	7-year incidence of HCC in patients with Hepatitis B treated with nucleos(t)ide analogues vs. untreated patients.	21,595 treated and 21,595 untreated	Nucleos(t)ide analogue	HCC diagnosis confirmed by pathology or imaging.	7 years	Antiviral therapy is associated with reduced risk of HCC in patients with chronic Hepatitis B.
Gao et al., 2014 [126]	Retrospective, multicenter cohort study of chronic Hepatitis B patients treated with Entecavir or Tenofovir.	275	Entecavir vs. Tenofovir	Viral suppression	18 months	In Hepatitis B surface antigen-positive patients, Tenofovir is significantly more effective in achieving complete viral suppression.
Ulcickas Yood et al., 2007 [118]	Retrospective cohort study comparing non-Hodgkin’s lymphoma incidence between chronic Hepatitis B patients and the general population.	3888	None	Incidence of non-Hodgkin’s lymphoma	7 year window	Chronic Hepatitis B patients are almost 3 times more likely to develop non-Hodgkin’s lymphoma.

## Data Availability

All data used can be found within the National Library of Medicine PubMed database. No new data was created.
